# Between Rigor and Relevance: Why the EU HTA Guidelines on Indirect Comparisons Miss the Mark

**DOI:** 10.3390/jmahp14020030

**Published:** 2026-05-07

**Authors:** Samuel Aballéa, Mondher Toumi, Piotr Wojciechowski, Emilie Clay, Bruno Falissard, Steven Simoens, Pascal Auquier, Stefano Capri, Renato Bernardini, Joerg Ruof, Frank-Ulrich Fricke, Oriol Sola Morales, Laurent Boyer

**Affiliations:** 1 InovIntell, 3023 GJ Rotterdam, The Netherlands; 2 CEReSS/UR3279—Health Services Research and Quality of Life Center, Aix-Marseille University, 13385 Marseille, France; mto@inovintell.com (M.T.); pascal.auquier@univ-amu.fr (P.A.); laurent.boyer@univ-amu.fr (L.B.); 3 Clever-Access, Wadowicka 8A, 30-415 Kraków, Poland; piotr.wojciechowski@clever-access.com; 4 Clever-Access, 75008 Paris, France; emilie.clay@clever-access.com; 5 CESP, INSERM U1018, Université Paris-Saclay, 94800 Villejuif, France; bruno.falissard@gmail.com; 6 Department of Pharmaceutical and Pharmacological Sciences, KU Leuven, 3000 Leuven, Belgium; steven.simoens@kuleuven.be; 7 School of Economics and Management, Cattaneo-LIUC University, 21053 Castellanza, Italy; scapri@liuc.it; 8 Department of Biomedical and Biotechnological Sciences (BIOMETEC), Section of Pharmacology, University of Catania, 95124 Catania, Italy; bernardi@unict.it; 9 European Access Academy, 4059 Basel, Switzerland; joerg.ruof@euaac.org; 10 Department of Business Administration, Georg Simon Ohm University of Applied Sciences Nuremberg, Bahnhofstraße 87, 90402 Nuremberg, Germany; frank-ulrich.fricke@th-nuernberg.de; 11 Health Innovation Technology Transfer, S.L. Carrer d’Aragó 60, pral 1a, 08015 Barcelona, Spain; osola@hittbcn.com

**Keywords:** European Union health technology assessment (EU HTA), joint clinical assessment (JCA), indirect treatment comparison (ITC), network meta-analysis (NMA), population-adjusted indirect comparisons (PAICs), matching-adjusted indirect comparison (MAIC), simulated treatment comparison (STC)

## Abstract

Indirect treatment comparisons (ITCs) are essential in the context of joint clinical assessments (JCAs) under Regulation (European Union [EU]) 2021/2282, bridging evidence gaps where head-to-head data are lacking and enabling assessment across diverse national patient, intervention, comparator, and outcome (PICO) requirements. This paper critically reviews the EU Health Technology Assessment Coordination Group’s (HTACG) guidelines on direct and indirect comparisons, with particular focus on ITCs. While the guidelines promote transparency and rigorous evaluation of assumptions, they adopt a restrictive stance on assumption violations, the use of unanchored comparisons, and population-adjusted methods such as matching-adjusted indirect comparisons (MAIC) and simulated treatment comparisons (STC). The guidance shows limited support for Bayesian methods and undervalues meta-regression in favor of subgroup analyses. Operational implications for health technology developers (HTDs) are substantial, including new requirements for dual systematic reviews, multiple network structures, and shifted null hypothesis testing. Moreover, the guidelines effectively dissuade the use of non-randomized comparisons in rare or rapidly evolving indications and may inadvertently hinder access to effective treatments. Emerging practices such as external control arms (ECA) or target trial emulation are underdeveloped. Notably, there is no indication that the guidelines are grounded in systematic methodological validation studies. As JCAs evolve, greater methodological flexibility, empirical grounding, and clear operational guidance will be essential. Refining the guidelines along these principles would enhance their practical utility, mitigate intrinsic assessment variability, support consistent assessments across Member States (MS), and ultimately improve patient access to innovative therapies.

## 1. Introduction

The implementation of Joint Clinical Assessments (JCAs) under Regulation (European Union [EU]) 2021/2282 marks a significant transformation in the landscape of health technology assessment (HTA) within the EU [1]. Central to this framework is the need to evaluate the certainty of the relative clinical effectiveness of new health technologies across a diversity of national healthcare contexts [1,2]. A distinctive feature of JCAs is the multiplicity of PICO (Population, Intervention, Comparator, Outcome) framework questions, reflecting the varied clinical practices, treatment comparators, outcomes of interest, populations and subgroups relevant across Member States (MS) [3,4,5,6]. This complexity presents considerable methodological challenges for comparative effectiveness research [7]. A recent exercise by the JCA subgroup identified 13 PICOs for two oncology indications [8].

In this setting, indirect treatment comparisons (ITCs) are an indispensable analytical tool. In many instances, head-to-head randomized controlled trials (RCTs) comparing the intervention with all relevant national comparators are not available at the time of assessment. As a result, robust and transparent methodologies for indirect evidence synthesis are essential to fill the evidence gaps and support HTA and reimbursement decision-making across jurisdictions.

The methodological importance of ITCs has been acknowledged in previous HTA frameworks, which refer to rigorous guidance for performing network meta-analyses (NMAs) and Population-Adjusted Indirect Comparisons (PAICs) in the absence of direct evidence. The most well-known and influential guidelines on this topic are probably those by the National Institute for Health and Care Excellence (NICE), Decision Support Unit (DSU) and the International Society for Pharmacoeconomics and Outcomes Research (ISPOR) Task Force [9,10,11]. The recent guidelines issued by the HTA Coordination Group (HTACG) extend this tradition by specifying harmonized methodological standards for the conduct and assessment of quantitative evidence synthesis within JCAs [12,13].

However, the HTACG guidelines also raise significant concerns related to the validity of ITCs. These guidelines explicitly require the verification of key assumptions such as similarity, homogeneity, and consistency to justify the validity of synthesis of evidence from multiple trials [12,13]. These assumptions are often difficult to meet in practice, particularly in the case of disconnected networks or when effect modifiers are unevenly distributed across studies. The increasing use of advanced methods, such as Matching-Adjusted Indirect Comparisons (MAIC), Simulated Treatment Comparisons (STC), and Multilevel Network Meta-Regression (ML-NMR), has further complicated the evidentiary landscape, calling for careful scrutiny of methodological rigor, transparency, and robustness [14]. To date, there is no clear definition of methods that can be universally and reliably applied to assess the value of products.

This paper provides a critical review of the HTACG’s methodological and practical guidelines on direct and indirect comparisons, with particular emphasis on indirect evidence synthesis. It highlights areas of ambiguity or methodological contention and evaluates their applicability in the JCA context. Special attention is given to the implications of these guidelines for health technology developers (HTDs).

## 2. Summary of the Guidelines

Overall, the HTACG promotes a conservative, hypothesis-driven approach to evidence synthesis. Direct comparisons using robust RCT data are preferred, and anchored ITCs are acceptable if all methodological requirements are met. When PAICs or unanchored comparisons are necessary, their use should be carefully justified, transparently reported, and subjected to extensive sensitivity analysis. The guidelines emphasize the importance of pre-specified analytical plans, expert statistical input, and critical evaluation of residual uncertainties throughout the synthesis process [12,13].

### 2.1. Key Assumptions

All forms of evidence synthesis, whether direct or indirect, are based on the fundamental assumption of exchangeability, which is achieved through three essential criteria [12,14,15,16,17]:

- Similarity of studies with respect to effect modifiers.
- Homogeneity of relative treatment effects across trials comparing the same interventions; and
- Consistency between direct and indirect comparisons within an evidence network.

Violation of any of these conditions undermines the validity of the analysis [18]. Similarity, in particular, is highlighted as difficult to verify in practice, given the potential presence of unobserved or even unknown effect modifiers [12].

### 2.2. Direct Comparisons

Direct comparisons are typically synthesized using standard pairwise meta-analysis methods [19]. Fixed-effect models (e.g., inverse variance or Mantel–Haenszel methods) are acceptable when the assumption of a common effect is tenable, although this requires strong justification [12]. In most cases, random effects models are preferred because of the expected heterogeneity between studies. The Knapp–Hartung method with the Paule–Mandel estimator is recommended when at least five studies are available; for fewer studies, the use of variance correction, the DerSimonian–Laird (DSL) method, qualitative summaries, or the alternative use of Bayesian methods with weakly informative priors are recommended [12].

### 2.3. Indirect Treatment Comparisons

In the absence of head-to-head trials, or when multiple interventions need to be evaluated simultaneously, indirect comparisons are necessary. The guidelines categorize ITC methods into three primary types [12]:

#### 2.3.1. Anchored Indirect Comparisons

These preserve randomization by estimating relative treatment effects using a common comparator. The Bucher method is appropriate for comparisons with a common comparator and can be applied to simple star networks [20]. The NMA is required for more complex evidence networks [17]. Both frequentist and Bayesian implementations of NMA are acceptable, with the latter offering advantages in handling sparse data and incorporating prior knowledge [12]. Validity depends on the assumptions of similarity, homogeneity, and consistency, all of which must be rigorously evaluated. Tools such as node splitting [21] and inconsistency models [22] can be used to formally evaluate inconsistency within closed loops.

#### 2.3.2. Population-Adjusted Indirect Comparisons

When the similarity assumption is not met, due to observed imbalances in effect modifiers, PAICs may be considered [12]. These require access to individual patient-level data (IPD) for at least one study and include methods such as MAIC, STC, and ML-NMR [14]. These approaches rely on the assumption of conditional constancy of relative effects, i.e., that all relevant effect modifiers have been correctly identified and included. Because of their complexity and sensitivity to modeling choices, guidelines recommend that PAICs be prespecified and accompanied by sensitivity analyses, including formal tests against shifted null hypotheses to account for residual uncertainty [12,13].

#### 2.3.3. Unanchored Comparisons and Non-Randomized Evidence

In disconnected networks without a common comparator, analyses are equivalent to comparisons based on non-randomized evidence. Such comparisons require full access to IPD and rigorous adjustment for confounding [23]. The guideline briefly acknowledges advanced IPD-based methods for confounding adjustment, including multiple regression, instrumental variables, and g-computation [12], but does not elaborate on their implementation or endorse them as standard practice. Instead, it emphasizes propensity score-based methods, such as matching or inverse probability weighting. These require that all known relevant confounders be measured and that assumptions of positivity, overlap, and covariate balance be satisfied [14]. Even when these conditions are met, results from non-randomized data are considered to have a higher uncertainty and risk of bias, and should be interpreted with caution [12]. Unanchored MAICs and STCs that use aggregate comparator data rather than IPDs are considered insufficient.

## 3. Critical Review

The HTACG guidelines on direct and indirect comparisons set an ambitious standard for methodological rigor in the JCA framework, going beyond those previously set by the NICE DSU, the ISPOR Task Force on ITC, or the Cochrane Handbook [9,10,11,24].

However, a critical analysis reveals several problematic areas where the guidelines are either overly conservative, inconsistently reasoned, or insufficiently aligned with accepted best practice in evidence synthesis. These are outlined in Text Box 1 and further detailed below.

Box 1 **Key limitations identified in the EU HTA Guidelines on quantitative evidence synthesis.**

- Overly conservative stance on assumption violations, risking the blanket rejection of indirect treatment comparisons (ITCs).
- Lack of clarity on the conditions for combining direct and indirect evidence; although NMAs are mentioned, the guidance may discourage broader network structures and mixed treatment comparisons. Restrictive preference for subgroup analyses over meta-regression, despite lower statistical power and greater risk of false positives.
- Limited endorsement of population-adjusted methods (e.g., MAIC, STC), despite their relevance when effect modifiers are imbalanced.
- Minimal consideration of Bayesian approaches, despite their strengths, particularly in sparse- or rare-event settings.
- Dissuasion of unanchored comparisons, overlooking recent advances in causal inference frameworks and evolving methods to quantify biases.
- No indication that recommendations are grounded in empirical validation or simulation studies.

### 3.1. Ambiguity in the Role of Indirect Evidence Alongside Direct Comparisons

The guidelines are unclear about whether indirect evidence can complement direct comparisons. While established methods such as NMA allow the integration of both evidence types, subject to a consistency check, the HTACG does not explicitly support this approach. The methodological guideline states, for example, “when treatments have not been directly compared in RCTs, indirect comparisons can be used” [12]. This formulation is ambiguous and might be interpreted as “when treatments have been directly compared, indirect comparisons cannot be used.”

The guidelines include passages that support the use of network meta-analysis when multiple pairwise comparisons are available. However, they also caution that “the inclusion of additional comparators and studies beyond those required to connect the network should generally be avoided, unless there is strong evidence to suggest that their inclusion improves certainty of evidence” [12]. This conditional statement is ambiguous. For example, in a widely cited antidepressants network meta-analysis [25], the inclusion of indirect evidence enhanced interpretability and yielded clinically meaningful distinctions between treatments. Yet one could still dispute whether this represented ‘strong evidence’ of increased certainty, given the accompanying heterogeneity. This illustrates how the guideline’s wording risks divergent interpretations, and why clearer encouragement of indirect evidence integration is warranted.

The EU HTA guideline effectively reverses the usual evidentiary paradigm [26]. Whereas existing guidance (NICE DSU, ISPOR, Cochrane [24]) presumes that all relevant evidence—direct and indirect—should be included unless its exclusion is justified, the EU HTA document requires positive proof that indirect evidence increases certainty before it can be incorporated [27,28,29]. This inversion places a higher bar on the use of indirect evidence, potentially limiting comprehensive synthesis. Discouraging the use of larger networks stands in opposition to the principle that broader networks, incorporating (more) indirect evidence, yield more precise estimates and support more generalizable inferences, when used appropriately [30].

### 3.2. Overly Restrictive Interpretation of Assumption Violations

The HTACG’s strict stance on assumption violations is a central concern. The methodological guideline asserts that “if any of these properties [similarity, homogeneity, consistency] do not hold, the results of an anchored indirect comparison are unlikely to provide a meaningful estimate” [12]. This fails to recognize the role of methods such as random-effects models, meta-regression, and sensitivity analyses in mitigating these limitations. NICE DSU guidance and other international sources promote such techniques as a standard part of evidence synthesis [28,31]. The HTACG also recommends the use of random-effects models, which are specifically designed to incorporate the uncertainty due to between-study heterogeneity into the confidence intervals. Yet this general statement imposes a ‘double penalty’ when assumptions are violated, making assessors uncertain about whether results can be relied upon even when they capture the uncertainty due to heterogeneity.

### 3.3. Anchored Population-Adjusted ITCs—Overcautious Appraisal

The guidelines acknowledge the relevance of methods such as MAIC, STC and ML-NMR in the context of anchored comparisons, yet caution that they are “often more suitable as an exploratory analysis rather than as the primary analysis” [13]. The HTACG’s concern that “the number of methods and potential covariate combinations available to the modeler raises the possibility of selecting the method that produces the most favorable results for the intervention under assessment” is understandable [13]. However, a population-adjusted indirect comparison (PAIC) is in principle less biased than an unadjusted analysis if well conducted [32]. This has been confirmed in simulation studies: Phillippo et al. (2018) [33] showed that unadjusted anchored ITCs were biased when covariates were imbalanced, while MAIC and STC substantially reduced this bias, although at the cost of higher variance. Remiro-Azocar et al. (2021) [34] extended this work to more complex scenarios and confirmed that population-adjusted methods outperformed unadjusted ITCs, particularly when effect modifiers had strong effects. These findings suggest that adjusted analyses should not be relegated to a purely secondary role, as long as assumptions are transparently reported and sensitivity analyses are performed. Although it is generally preferable to use IPD for at least one study to adjust for differences in population characteristics, adjustments using aggregate data only (e.g., aggregate-level meta-regression) can also reduce bias relative to unadjusted analyses in some settings.

An important limitation of anchored MAIC and STC analyses is that resulting estimates represent the treatment effect in the population of the comparator study, which may differ from the target population. However, another PAIC method, namely ML-NMR, can be used to predict treatment effects in a pre-specified target population, representing the actual decision-relevant population [35].

While HTACG raises concerns about the validity and relevance of PAICs, it is important to note that these limitations are not unique to PAICs. All ITCs—including Bucher comparisons and NMAs—are subject to potential bias if relevant effect modifiers are missing, and their results are conditional on the populations included in the contributing trials. This is why international evidence appraisal frameworks such as GRADE highlight “indirectness” as a general limitation of indirect evidence [36]. Singling out PAICs risks overstating their limitations relative to other indirect comparison methods. The guideline uniquely recommends applying shifted null hypothesis testing to PAICs to account for possible unmeasured effect modifiers, without requiring the same for standard NMA or Bucher methods [12]. All indirect comparisons, adjusted or unadjusted, are subject to unverifiable assumptions about effect modifiers [11]. If this approach of shifted null hypothesis testing is considered informative, it is unclear why it would apply only in the case of PAICs.

If bias is suspected, one would typically interpret the results qualitatively with caution or adjust the level of certainty (e.g., GRADE “very low certainty” if there is a high risk of bias). Imposing a formal test threshold Δ could be seen as an arbitrary second hurdle. For instance, if a hazard ratio is 0.85 with 95% CI 0.72–0.99 (*p* < 0.05 for H0:1), a shifted null approach might say “we will only consider it significant if *p* < 0.05 for H0:0.90”—effectively requiring the CI to exclude 0.90. Why 0.90? The guideline offers no specific literature, leaving it to the HTD to justify. Furthermore, the guideline explicitly defers threshold setting to MS, which somewhat contradicts the idea of a unified assessment. France’s Haute Autorité de Santé (HAS) and Germany’s Institut für Qualität und Wirtschaftlichkeit im Gesundheitswesen (IQWiG) remain highly resistant to ITC evidence [37,38,39,40], while Sweden, Netherlands, Norway, and Belgium show greater openness to such data [38,41]. Allowing MS to set their own thresholds enables them to accept or reject ITC results based on their preferences. This does not align with JCA’s goal to standardize clinical assessment between countries.

Although the HTACG does not strictly mandate shifted null hypothesis testing, it does in effect endorse the approach by mentioning it multiple times, including in “key points” and in the summary, while alternatives are cursorily mentioned. Other published approaches such as E-values [42], mentioned once as an “other method” in the guidelines, and quantitative bias analysis (QBA) [43], never explicitly mentioned, are available. Highlighting the shifted null hypothesis approach is inconsistent with the stated objective of the methodological guideline, which is to describe the “most commonly used” methods for ITC. Furthermore, while the threshold in shifted null hypothesis testing lacks a clear clinical interpretation, the QBA method explicitly quantifies the potential impact of unmeasured confounding. Within this framework, E-values provide a straightforward summary: they indicate the minimum strength of association that an unmeasured confounder would need to have with both the treatment and the outcome to fully explain away an observed effect. This translation of statistical uncertainty into a clinically meaningful metric makes it easier for assessors and clinicians to judge plausibility.

### 3.4. Meta-Regression vs. Subgroup Analyses—Misplaced Prioritization

Another controversial recommendation is the endorsement of subgroup analyses over meta-regression. While the methodological guideline recognizes that both subgroup analysis and meta-regression can be used, it explicitly states that “in the context of JCA, subgroup analyses are often more useful than meta-regression” [12]. Subgroup analyses may be more widely applicable, but the wording chosen by HTACG risks undermining the value of meta-regression in the eyes of assessors. Subgroup analyses, especially those based on aggregate trial-level data, are underpowered, prone to spurious findings, often require dichotomization of continuous variables, and sometimes violate randomization. In contrast, meta-regression, when applied appropriately, can exploit continuous covariates across trials and detect trends that would be missed by binary subgroup splits [44].

This contradicts the view presented in the NICE DSU TSD 3, which emphasizes the use of meta-regression where feasible and only warns against ecological bias, without suggesting that one method is inherently superior [28]. A more balanced approach would advocate for a case-by-case evaluation, with sensitivity analyses using both methods where appropriate.

### 3.5. Underappreciation of Bayesian Methods

Bayesian approaches are described merely as “alternative” options to the frequentist norms and are conditioned on the availability of priors [13], despite being acknowledged as especially advantageous in the small-sample and rare-event scenarios [45]. For example, the practical guideline details frequentist methods for meta-analysis and notes that Knapp–Hartung methods may yield “misleadingly narrow” CIs in homogeneous settings and “non-informative results” when data are sparse, before noting that Bayesian methods can be used as an “alternative” [13]. By contrast, the widely accepted literature endorses Bayesian methods as preferred in sparse or uncertain settings because of their ability to incorporate prior knowledge and produce interpretable posterior distributions [46,47,48].

Furthermore, the HTACG guidelines may result in assessment dossiers employing a mix of frequentist and Bayesian methods across different comparators or outcomes, depending on data availability and the specific context. While acknowledging data limitations, this approach can introduce complexity. Specifically, combining frequentist and Bayesian methods within a single assessment (i.e., mixing 95% confidence intervals and 95% credibility intervals; *p*-values and posterior probabilities) can pose challenges to maintaining a consistent inferential framework. This could lead to difficulties in the interpretation of results, and conflict with the Neyman–Pearson principles that underlie much of frequentist hypothesis testing.

We argue that the choice between frequentist and Bayesian approaches should not be made purely on pragmatic grounds (e.g., adopting Bayesian methods only when frequentist methods are limited in small-sample contexts). Instead, method selection should follow a prespecified decision framework that ensures interpretability and coherence across outcomes. Without such a framework, dossiers risk producing non-comparable inferential statements, undermining transparency [49]. Moreover, given the complexities associated with applying frequentist multiplicity controls in the diverse landscape of HTA, a consistent application of a Bayesian framework may offer a more coherent and interpretable approach to conducting ITCs.

### 3.6. Unanchored Comparisons and Non-Randomized Evidence—An Overly Restrictive Stance

The HTACG takes a strong negative stance on unanchored indirect comparisons, stating that “only anchored indirect comparisons are appropriate, as these respect within-study randomization” [12]. While preserving randomization is methodologically ideal, this stance against unanchored methods is both impractical and inconsistent with modern HTA practice [50]. Single-arm trials (SATs) are increasingly used, especially in oncology and rare diseases. Between 2002 and 2021, approximately 31% of United States Food and Drug Administration (FDA) oncology approvals were based solely on SATs [51]. The European Medicines Agency (EMA) also recognizes that SATs may serve as pivotal evidence in specific regulatory contexts [52].

The guidelines risk discouraging informative analyses in situations where no other comparative evidence is available. Unanchored comparisons rely on strong assumptions and are highly sensitive to residual confounding; however, their credibility can be assessed on a case-by-case basis using quantitative sensitivity/bias analyses (e.g., QBA, E-values) alongside comprehensive reporting of assumptions, rather than labelling them as generally unreliable. Despite their limitations, unanchored MAIC and STC also have a role to play in informing decision-making [50]. The guidelines’ dissuasion of unanchored comparisons thus contrasts with a growing international consensus that, while such methods carry higher uncertainty, they may still support decision-making when carefully designed and transparently reported. This stance also conflicts with EBM principles, which advocate using all available meaningful evidence.

Although the HTACG considers unanchored comparisons to be generally “inappropriate”, the guidelines do provide a succinct description of relevant methods [12,13]. When the pivotal evidence for the assessed product is based on an SAT, the guidelines stipulate that “adjustment methods require access to the full IPD”, for both the assessed product and the comparator. This effectively positions MAICs or STCs, which utilize aggregate comparator data, as less desirable than ECAs. This is a contentious point. In practice, IPD are often unavailable for comparator trials, necessitating the use of ECAs derived from retrospective real-world data [53]. Consequently, the choice between an ECA based on potentially lower-quality real-world data and a MAIC or STC using higher-quality aggregate data is often complex, and the guidelines’ preference for ECAs in this context is debatable.

### 3.7. Overlooked Considerations in Living Evidence Synthesis

While the guideline references the need for evidence synthesis to be current, it omits any discussion of living NMA or updating mechanisms [12]. This is a missed opportunity, particularly in fast-evolving fields like oncology, where evidence landscapes shift rapidly. Cochrane and other methodological leaders now emphasize living NMAs as essential for maintaining relevance and responsiveness to emerging data [54].

### 3.8. Support for Advanced Time-to-Event (TTE) Methods

The guideline’s handling of TTE data is more balanced. It rightly calls for testing the proportional hazards (PH) assumption and recommends alternatives such as restricted mean survival time (RMST) and flexible survival models when PH does not hold. This is consistent with emerging best practices, particularly in oncology where PH violations are common [55].

## 4. Implications for JCA Submissions

The HTACG’s guidelines on direct and indirect comparisons impose significant methodological and procedural expectations that have substantial implications for HTDs preparing JCA submissions. The reporting requirements for ITCs go well beyond traditional HTA standards.

First, the guidelines emphasize the need for prespecified strategies and transparent criteria to identify covariates that may impact relative treatment effects. More specifically, HTDs must now conduct not only a systematic literature review (SLR) to identify clinical evidence on treatment effects, but also a second comprehensive review to identify potential effect modifiers, as well consultations with health care professionals (HCPs) with experience in the disease area [12,13]. The implication is that reliance on subgroup analyses identified in the clinical study report and SLR and consultations with clinicians employed by the HTD will no longer suffice. The need to formally involve HCPs with disease-specific expertise in identifying effect modifiers adds further operational complexity, as this consultation must presumably be documented and justified within the submission.

Second, the guidelines mandate that JCA dossiers include both population-level and comparator-level evidence networks when conducting NMAs [13]. While this may enhance transparency for national decision makers, it introduces an analytical burden that could be significant for submissions with multiple comparators. HTDs will need to construct and validate several network structures, increasing the risk of type I error and complicating consistency evaluations. No empirical justification is provided for this dual approach, which may lead to duplication of effort without corresponding benefit.

Third, the guideline’s preference for shifted null hypothesis testing in adjusted ITCs (e.g., MAIC/STC) imposes an additional evidentiary hurdle. HTDs are expected to defend the choice of a clinically meaningful threshold (e.g., a relative risk reduction of 10%) without a standard benchmark, leaving room for arbitrary or inconsistent assessments across MS. This creates uncertainty about how population-adjusted estimates will be received and whether they will be discounted based on subjective criteria.

Fourth, the guidelines recommend that prediction intervals, not just confidence or credible intervals, be routinely reported in random-effects models. This requirement is methodologically sound, but rarely fulfilled in current reports of ITCs [56]. HTDs must now plan to generate and interpret prediction intervals, particularly for sparse data scenarios where between-study heterogeneity may be high.

Fifth, while the use of unanchored comparisons is generally discouraged, there is a clear preference for ECAs over PAIC using aggregate data for the comparator, such as MAICs and STCs. This stance diverges from recent HTA trends: many relative effectiveness assessments based on SATs, especially in oncology, have relied on MAICs and STCs, while the use of formal ECAs remains relatively rare in European regulatory and HTA contexts [53,57]. Recent empirical reviews have found that MAIC and STC methods are commonly used and accepted in health technology submissions when SATs serve as pivotal evidence, largely due to the IPD requirement being limited to sponsor-held data [53]. In contrast, ECAs requiring either high-quality observational data or access to relevant patient registries pose significant feasibility challenges in terms of data access, endpoint alignment, and methodological transparency [58,59]. Nonetheless, as real-world data infrastructures mature and the methodological landscape evolves, HTDs should increasingly consider the feasibility of developing ECAs in such contexts. Approaches such as target trial emulation, leveraging observational datasets with robust confounding adjustment (e.g., propensity scores or doubly robust estimators), may offer a viable pathway toward more acceptable unanchored evidence [60,61].

Given the multiplicity of PICO questions [3,4,5,8] inherent in the JCA process and the need for sensitivity analyses and subgroup analyses, these expectations cumulatively lead to a marked increase in analytical and submission workload. In sum, HTDs will need to allocate additional resources—statistical, clinical, and procedural—to ensure compliance with the HTACG’s expectations. These guidelines not only raise the methodological bar, but also introduce substantial practical complexity, which, if not addressed proactively, could jeopardize the acceptability of the indirect evidence submitted in support of new interventions.

## 5. Conclusions

The HTACG’s methodological and practical guidelines on ITCs represent an important step towards harmonizing evidence synthesis practices across Europe within the JCA framework. The guidelines promote transparency, detailed reporting, and rigorous assessment of key assumptions—principles that are essential for robust comparative effectiveness assessments in an increasingly complex healthcare landscape [12,13].

However, this review has identified several important limitations that warrant careful consideration. First, the guidelines take an overly conservative stance toward assumption violations in ITCs, with a tendency to encourage rejection of analyses rather than consideration of available adjustment methods. Techniques such as meta-regression, population-adjusted indirect comparisons, and Bayesian modeling are acknowledged, but often relegated to secondary or exploratory roles, despite their widespread acceptance in international HTA and regulatory contexts [37,38].

Second, the guidelines do not adequately address the integration of direct and indirect evidence, nor do they clearly support the use of mixed treatment comparisons when both types of evidence are available. In areas where assumptions may not fully hold, such as the presence of unknown effect modifiers or missing covariates, the recommendations favor study exclusion over analytical adjustment, potentially leading to loss of evidence and biased conclusions.

Third, while the guidelines express methodological caution about unanchored comparisons, they do not adequately recognize the practical realities that necessitate such approaches, particularly in rare diseases and oncology, where SATs remain prevalent [62]. The lack of detailed guidance on acceptable methods for ECAs and on strategies such as target trial emulation represents a missed opportunity to promote more sophisticated use of real-world evidence in HTA [61].

Fourth, the guidance introduces several operational requirements for HTDs, including dual systematic reviews, comparator-level network analyses, and shifted hypothesis testing, that significantly raise the complexity of JCA submissions. While these requirements may enhance methodological thoroughness, they risk reducing the feasibility and efficiency of submissions, considering the short timelines [63], particularly for smaller companies or in therapeutic areas with fragmented evidence bases.

Finally, it is worth noting that the developers of the HTACG guidelines do not appear to have adhered to the same evidence-based standards that they recommend for submissions. The guidelines lack transparent documentation of the evidence base underpinning their methodological choices. Specifically, there is no mention of results from empirical evaluations or simulation studies that assess the performance, validity, or limitations of different evidence synthesis and ITC approaches. This omission raises concerns about the robustness and transparency of the recommendations. Best practice in guideline development, such as that advocated by GRADE [64], emphasizes the importance of grounding recommendations in a systematic review of methodological evidence.

Despite the EU HTA guidelines’ aim to create a unified methodological framework, it is doubtful that they will have a practical impact on harmonizing the perception and utilization of ITCs across Europe. Historically, HTA agencies have exhibited markedly divergent attitudes toward indirect evidence. In Germany, IQWiG has set stringent requirements for indirect comparisons, making submissions of ITCs challenging to implement in practice [65]. Also, IQWiG rejected 94% of ITCs submitted in benefit assessments between 2011 and 2017, largely due to concerns about study suitability, similarity, and statistical methods [66]. Similarly, the French Haute Autorité de Santé (HAS) has maintained a cautious stance on accepting adjusted indirect evidence. The current EU HTA guidelines risk reinforcing these conservative positions by providing formal arguments for rejecting analyses without offering mechanisms to promote alignment or mutual recognition of evidence standards across Member States. The development of a framework to grade the certainty of indirect evidence could be part of such harmonization mechanisms, whereas the current guidelines tend to dismiss evidence in a binary manner.

Guidelines alone are unlikely to address the concern feeding the resistance of some HTA agencies against indirect evidence. Randomized controlled trials (RCTs) remain the gold standard for clinical evidence, in part because all analytical methods are prespecified in study protocols and statistical analysis plans, limiting the scope for post hoc methodological choices. In contrast, ITCs—especially those requiring population or outcome adjustments—are often conducted after trial completion, making full pre-specification difficult, as the methodology needs to be adapted according to available data. Also, the suspicion surrounding ITCs may stem less from flaws in the methodologies themselves than from the flexibility they afford health technology developers (HTDs) in selecting analytic strategies that may yield more favorable outcomes. Nonetheless, ITCs are indispensable in the health technology assessment (HTA) process: it is neither feasible nor ethical to conduct head-to-head RCTs for every comparator in every population of interest. If the EU HTA regulation is to facilitate access to innovative therapies across MS, it must allow for PICO questions to be answered through rigorously conducted and transparently reported ITCs, and HTA agencies must accept adjusted analyses. If concerns about selective reporting persist, greater involvement of independent agencies—either through independent conduct of ITCs or supervisory roles during their design and analysis—may be necessary to ensure both credibility and acceptability of adjusted indirect evidence. As those agencies may not be able to access IPD and therefore may not be able to conduct ITCs directly, their role could be exercised through active supervision of the planning and implementation of analyses.

In conclusion, while the HTACG guidance documents establish a rigorous foundation for evidence synthesis in the EU, their current formulation may inadvertently constrain methodological flexibility and limit the practical use of valid ITCs. To ensure both scientific credibility and operational feasibility, future updates should aim to achieve the following:

- Provide clearer support for the integration of direct and indirect evidence (i.e., “mixed” treatment comparisons);
- Allow for appropriately adjusted analyses in the presence of assumption violations;
- Guide, rather than restrict, the cautious use of unanchored comparisons when no alternatives exist, including more guidance on the use of real-world evidence and ECAs;
- Enhance support for Bayesian methods: the HTACG may have given more attention to frequentists methods assuming that assessors are more familiar with them, although these guidelines provide in fact an opportunity to help assessors understand the value of Bayesian methods;
- Incorporate operational guidance that reflects the complexity and diversity of real-world submissions;
- Propose a framework for grading the strength of indirect evidence.

Addressing these issues through improvements suggested in Text Box 2 could enhance the guidelines’ utility, support more consistent and equitable HTA outcomes across MS, and ultimately improve timely patient access to effective therapies in the EU.

Box 2 **Suggested improvements for the EU HTA guidelines on indirect treatment comparisons.**

- Avoid reversing the evidentiary paradigm. The default approach should be to include all relevant direct and indirect evidence unless exclusion is justified, not to require positive proof that indirect evidence increases certainty.
- Consider the wider use of Bayesian methods, which are well suited to quantitative evidence synthesis; provide readers with the knowledge required to engage with Bayesian approaches; and reassess the appropriateness of combining Bayesian and frequentist analyses within the same dossier (currently allowed by the guidelines)
- Offer balanced recommendations on population-adjusted methods (e.g., MAIC, STC, ML-NMR), acknowledging their relevance as primary analyses in many situations, subject to the use of appropriate methods to quantify uncertainty. In anchored ITCs affected by treatment effect modifiers, ML-NMR would generally be preferable to other PAIC methods, as it can estimate treatment effects for a pre-specified target population, rather than only for the comparator study population.
- Explicitly acknowledge that all evidence has limitations, and propose a framework for grading the certainty of indirect evidence (e.g., in the manner of GRADE) to support decisions under imperfect evidence, potentially incorporating quantitative bias analysis.
- Expand guidance on leveraging real-world evidence and external control arms, including the adoption of emerging methods such as target trial emulation.
- Consider the practical feasibility of the guidelines, recognizing the limited time and resources available to both health technology developers and national assessors.
- Ground methodological recommendations in a systematic review of empirical validation studies and simulation research, ensuring that guidance reflects the best available evidence.

## Data Availability

No new data were created or analyzed in this study.

## Abbreviations

The following abbreviations are used in this manuscript:

CI	Confidence Interval
DSU	Decision Support Unit (of NICE)
Δ (Delta)	Symbol used for a threshold in hypothesis testing
EBM	Evidence-Based Medicine
ECA	External Control Arm
EMA	European Medicines Agency
EU	European Union
FDA	United States Food and Drug Administration
GRADE	Grading of Recommendations, Assessment, Development and Evaluation
H0	Null Hypothesis
HAS	Haute Autorité de Santé (France)
HCP	Health Care Professional
HTA	Health Technology Assessment
HTACG	Health Technology Assessment Coordination Group (of the EU)
HTD	Health Technology Developer
IPD	Individual Patient Data
IQWiG	Institut für Qualität und Wirtschaftlichkeit im Gesundheitswesen (Germany)
ISPOR	International Society for Pharmacoeconomics and Outcomes Research
ITC	Indirect Treatment Comparison
JCA	Joint Clinical Assessment
MAIC	Matching-Adjusted Indirect Comparison
ML-NMR	Multilevel Network Meta-Regression
MS	Member States (of the EU)
NICE	National Institute for Health and Care Excellence
NMA	Network Meta-Analysis
PAIC	Population-Adjusted Indirect Comparison
PH	Proportional Hazards
PICO	Population, Intervention, Comparator, Outcome
RCT	Randomized Controlled Trial
RMST	Restricted Mean Survival Time
SAT	Single-Arm Trial
SLR	Systematic Literature Review
STC	Simulated Treatment Comparison
TSD	Technical Support Document (from NICE DSU)
TTE	Time-To-Event
